# Quantification of endogenous and exogenous protein expressions of Na,K-ATPase with super-resolution PALM/STORM imaging

**DOI:** 10.1371/journal.pone.0195825

**Published:** 2018-04-25

**Authors:** Kristoffer Bernhem, Hans Blom, Hjalmar Brismar

**Affiliations:** 1 Science for Life Laboratory, Department of Applied Physics, Royal Institute of Technology, Stockholm, Sweden; 2 Science for Life Laboratory, Department of Women’s and Children’s Health, Karolinska Institutet, Stockholm, Sweden; J. Heyrovsky Institute of Physical Chemistry, CZECH REPUBLIC

## Abstract

Transient transfection of fluorescent fusion proteins is a key enabling technology in fluorescent microscopy to spatio-temporally map cellular protein distributions. Transient transfection of proteins may however bypass normal regulation of expression, leading to overexpression artefacts like misallocations and excess amounts. In this study we investigate the use of STORM and PALM microscopy to quantitatively monitor endogenous and exogenous protein expression. Through incorporation of an N-terminal hemagglutinin epitope to a mMaple3 fused Na,K-ATPase (α_1_ isoform), we analyze the spatial and quantitative changes of plasma membrane Na,K-ATPase localization during competitive transient expression. Quantification of plasma membrane protein density revealed a time dependent increase of Na,K-ATPase, but no increase in size of protein clusters. Results show that after 41h transfection, the total plasma membrane density of Na,K-ATPase increased by 63% while the endogenous contribution was reduced by 16%.

## Introduction

Super-resolution single-molecule localization microscopy [1–5], has in the last decade made it possible to visualize biological specimens on the nanoscale. By labeling an investigated protein with a fluorescent reporter, which can be made to switch between a fluorescently detectable state (active, ON) and non-detectable state (non-active, OFF), separation in time and space of single molecules becomes possible. The spatio-temporally separate labeled entities are then used to map out cellular topology with increased resolving power. To generate a full representation of the labeled sample, the stochastic switching and detection process is repeated many times (several thousand to tens of thousands frames are recorded). Finally, mathematical analysis of the detected fluorescence (i.e. pinpointing the location of the stochastically switching fluorescent molecules) is used to generate a reconstructed super-resolution image of the labelled sample [6, 7]. In addition to improving resolution, as compared to conventional microscopy where no separation of labelled molecules occurs, super-resolution single-molecule localization imaging allows for quantitative analysis [8–11]. Quantitative information of molecular coordinates can be used to extract protein densities, protein cluster sizes, and stoichiometry if applied accurately [12–15]. Prior to selection of imaging and analysis strategies to minimize possible misinterpretations of data quantification, protein labeling must be under essential control. As pointed out in the literature, both immunofluorescence labeling, and transfection can skew the quantification of protein densities, a challenge that has stimulated recent method development in labeling protocols for super-resolution imaging [16–18].

Overexpression of proteins and fusion-proteins in particular, through transient transfections provide an easy way of introducing a mutant protein or study the distribution of a protein in cells. The principal drawback of transient transfections is the degree of overexpression typically associated with it [19]. This can be alleviated in some cases as most proteins require interactions with other proteins to function properly and these can then act as limiting regulators for the system. Even for abundant proteins, such as G-protein coupled receptors, transient expression can reach several orders of magnitude above endogenous levels with changes in signaling behavior as a possible result [20]. In addition to the effects of an over-expressed protein amount, the cell will also be stressed by the transient over-expression with additional uncontrolled or unknown effects [21].

In this work we have through use of transient transfection, immunohistochemistry and super-resolution imaging quantified the plasma membrane protein density of Na,K-ATPase α_1_ (ATP1A1). Na,K-ATPase is a heteromeric protein, essential for maintaining cellular membrane potential and control of intracellular ion homeostasis. Na,K-ATPase has a catalytically active α-subunit that requires a β-subunit for assembly into a functional protein and plasma membrane insertion. Plasma membrane levels of such proteins should potentially be less affected by an overexpression as the interacting partner remains at endogenous levels [22, 23].

## Materials and methods

### Chemicals

All chemicals, unless explicitly stated, were purchased from Sigma Aldrich. Immunocytochemistry PBS (iPBS) (137 mM NaCl, 2.7 mM KCl, 10 mM NaH_2_PO_4_, 1.8 mM KH_2_PO_4_, 1 mM CaCl_2_ and 0.5 mM MgCl_2_, pH 7.40, sterile filtered) was used for washing of fixed cells. FlAsH-EDT2 was purchased from ThermoFischer Scientific.

### Cells

Human Embryonic Kidney (HEK) 293a cells (Invitrogen) cells were cultured according to supplier’s recommendations using DMEM (Life Technologies) with high glucose and pyruvate in 10% fetal bovine serum (Life Technologies). Cells were used between passage 3 and 10.

### Plasmids and transfection

HA_hNKAa1_mMaple3, a N-terminal fusion protein of hemagglutinin and mMaple3 [24] to human Na,K-ATPase α1 was synthesized by GenScript. hNKAa1_SEP, a superecliptic pHluorin (W317-SEP-L318)—human Na,K-ATPase α1 fusion protein, was cloned as previously described [25]. Mock transfection were performed using Syntaxin-TC, a plasmid encoding for a plasma membrane anchor, linker and a tetracysteine motif for FlAsH binding. Syntaxin-TC was synthesized by GenScript. All transfections were performed using lipofectamine LTX (Life Technologies) with 2 μg DNA following recommended protocols by the provider.

### FlAsH staining

HEK293a cells expressing Syntaxin-TC cultured on 18 mm round #1.5 coverslips (Marienfeld) were washed once in warm Opti-MEM (ThermoFischer Scientific) followed by staining using 2.5 μM of FlAsH-EDT2 for 60 min in incubator. The samples were then washed twice for 10 minutes in BAL wash buffer (1:100 dilution of BAL buffer in Opti-MEM) and subsequently fixed according to the STORM sample preparation below.

### PALM sample preparation

HEK293a cells expressing HA_hNKAa1_mMaple3 cultured in 35 mm round petri dishes with glass bottom (#1.5 coverslips) (MatTek) were fixed using ice cold 4% paraformaldehyde in iPBS for 10 min in +4°C. Samples were subsequently washed 3x10 min in iPBS and stored covered in iPBS in light protected container in +4°C until imaged.

### STORM sample preparation

HEK293a cells expressing HA_hNKAa1_mMaple3 cultured on 18 mm round #1.5 coverslips (Marienfeld) were fixed using ice cold MeOH for 10 min in +4°C. Samples were subsequently washed 3x10 min in iPBS. Samples were blocked for 60 min in room temperature using 10% bovine serum albumin in iPBS (filtered). Samples were stained using a 5 μg / ml concentration of the monoclonal anti Na,K-ATPase α1 antibody a6f (DSHB, University of Iowa) [26] for 60 min in room temperature. Alternatively, the samples were stained using a 5 μg / ml concentration of the monoclonal anti-HA antibody HA-7 (Sigma Aldrich). Samples were washed 3x10 min using iPBS followed by overnight staining using a 4 μg / ml concentration of anti-mouse Alexa647 (Invitrogen). Samples were washed 3x10 min and stored in light protected container in +4°C until imaged. Samples were mounted in a magnetic chamber (Chamlide) with the following STORM buffer. 1:100 GLOX 100x (10 mg glucose oxidase in 180 μl of 10 mM Tris (pH 7.0) and 10 mM NaCl) and 1:100 1 M MEA (77 mg cystamine in 1 ml 1:12 HCl) in 50 mM Tris-Cl (pH 8.0) 10 mM NaCl and 10% glucose. Cells were imaged within 60 minutes after application of buffer.

For imaging of Na,K-ATPase β1, HEK293a cells cultured on 18 mm round #1.5 coverslips (Marienfeld) were fixed using ice cold 10% trichloroacetic acid for 10 min in +4°C. Samples were subsequently washed 3x10 min in iPBS and permeabilized for 2 minutes using 0.25% Triton X-100 and subsequently washed for 3x5 min in iPBS. Samples were blocked for 60 min in room temperature using 10% bovine serum albumin in iPBS (filtered) (supplemented with 0.1% Triton X-100). Samples were stained using a 5 μg / ml concentration (supplemented with 0.1% Triton X-100) of β1 antibody (Santa-Cruz, sc21713) for 60 min in room temperature, which provided the best labelling efficiencies at concentrations above saturation. Samples were washed 3x10 min using iPBS followed by overnight staining using a 4 μg / ml concentration of anti-mouse Alexa647 (Invitrogen) in iPBS (supplemented with 0.1% Triton X-100). Samples were washed 3x10 min and stored in light protected container in +4°C until imaged. Samples were mounted in a magnetic chamber (Chamlide) with STORM buffer.

### PALM/STORM imaging

Single-molecule localization microscopy was performed using a Carl Zeiss Elyra PS.1 microscope equipped with 405-, 488-, 561- and 642-nm activation and excitation lasers. The objective used was a Plan-Apochromate 100x/1.46 Oil (Zeiss) and fluorescence was detected on a liquid cooled EMCCD camera (Andor Technology). Emission was collected through appropriate dichroic mirrors and bandpass filters set at 570–620 nm for detection of mMaple3, and 655 nm long pass filter for detection of Alexa647. For PALM imaging of mMaple3, activation was performed with 5% of 405 nm laser power (maximum input 10.1 mW), together with 100% laser power of 561 nm excitation (maximum input 57.8 mW). Illumination was performed over a 25x25 μm area in the sample (1/e^2^ spatial irradiance distance) in TIRF-mode. Single molecule fluorescence detection with the EMCCD camera was acquired with 100 x 100 nm pixel sizes, 20 ms integration time and 150 gain for mMaple3. 30 000 images were acquired for each cell imaged. The mean precision, σ in PALM measurements was 20±5 nm. For 2D-STORM imaging of Alexa647, back-pumping with 1.8% of 405 nm laser power was used together with 100% laser power of 642 nm excitation (maximum input power 25.4 mW). 30 000 images were acquired for each cell imaged and acquired with 100 x 100 nm pixel sizes, 20 ms integration time and 100 gain. For 3D-STORM imaging of Alexa647, with the inserted PRILM (phase ramp imaging localization microscopy) unit [27], 405 nm back-pumping power of 1% was used together with 100% of 642 nm excitation power. Cells were selected based on SEP fluorescence, detected by 488 nm excitation and 510–540 band pass filter. Single molecule fluorescence detection on the EMCCD camera was acquired with 100 x 100 nm pixel sizes, 25 ms integration time and 100 gain. The mean precision, σ in STORM measurements was 12±5 nm. 3D calibration for axial look-up coordinates was performed on 40 nm fluorescent beads, according to recommendations from the microscope manufacturer.

### Image analysis

**Fig 1:**
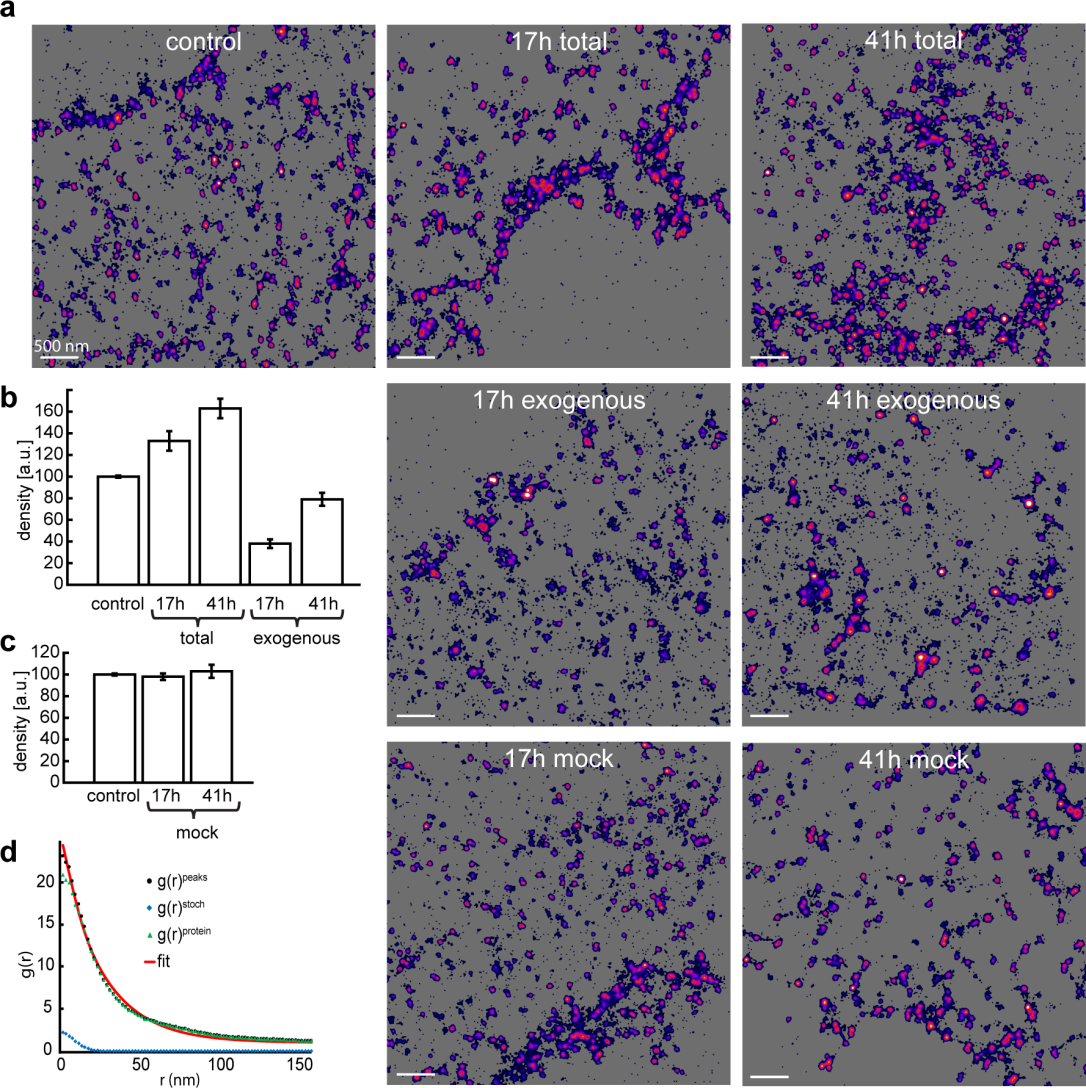
STORM imaging of Alexa647 stained HEK293a cells. (a) Representative rendered images from SMLM datasets based on: HEK293a cells (control), HEK293a cells transfected for 17 or 41 h with HA-hNKAa1_mMaple3 and stained using a6f antibody (total) recognizing both endogenous and exogenous Na,K-ATPase or anti-HA antibody (exogenous). Final two rendered images (mock) are of HEK293a cells transfected for 17 or 41 h with Syntaxin-TC. (b) Mean plasma membrane density (control, total and exogenous), n = 10–12 cells. Error bars show standard error of mean. All groups are significantly different at p < 0.05. (c) Mean plasma membrane density (control and mock), n = 5–12 cells. No significant difference at p < 0.05. Error bars show standard error of mean. (d) Representative pair correlation cluster analysis of a dataset transfected for 41h. Plot of the autocorrelation function for all detected events, g(r)^peaks^, the correlation due to multiple blinking events of a single molecule, g(r)^stoch^, and the corrected protein correlation g(r)^protein^ after a fit to to a model for clustered distribution of proteins.

**Fig 2:**
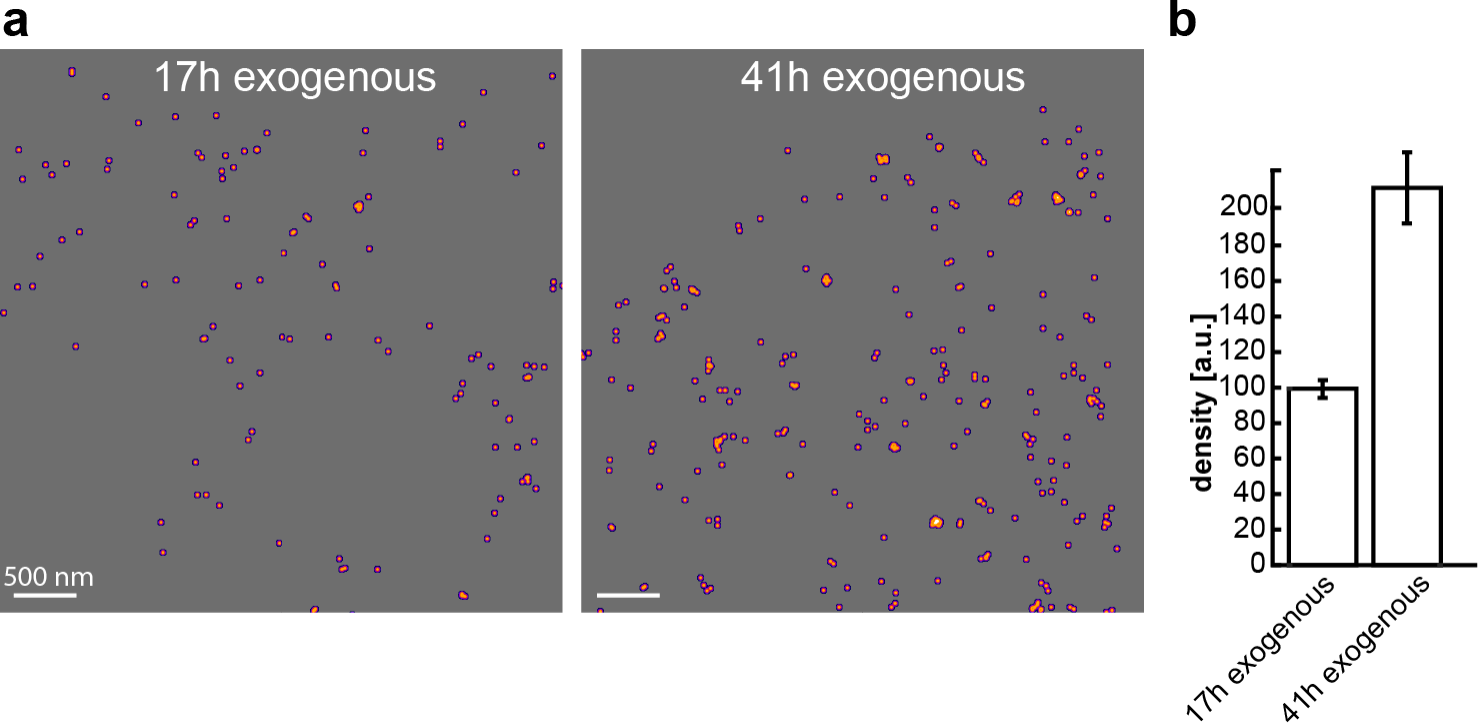
PALM imaging of exogenous α1-expression. **(**a) Representative rendered images from mMaple3 SMLM datasets based on HEK293a cells transfected for 17 or 41 h with HA-hNKAa1_mMaple3. (b) Mean plasma membrane density for exogenous expression, n = 17 or 27 cells, significantly different at p < 0.05. Error bars show standard error of mean.

**Fig 3:**
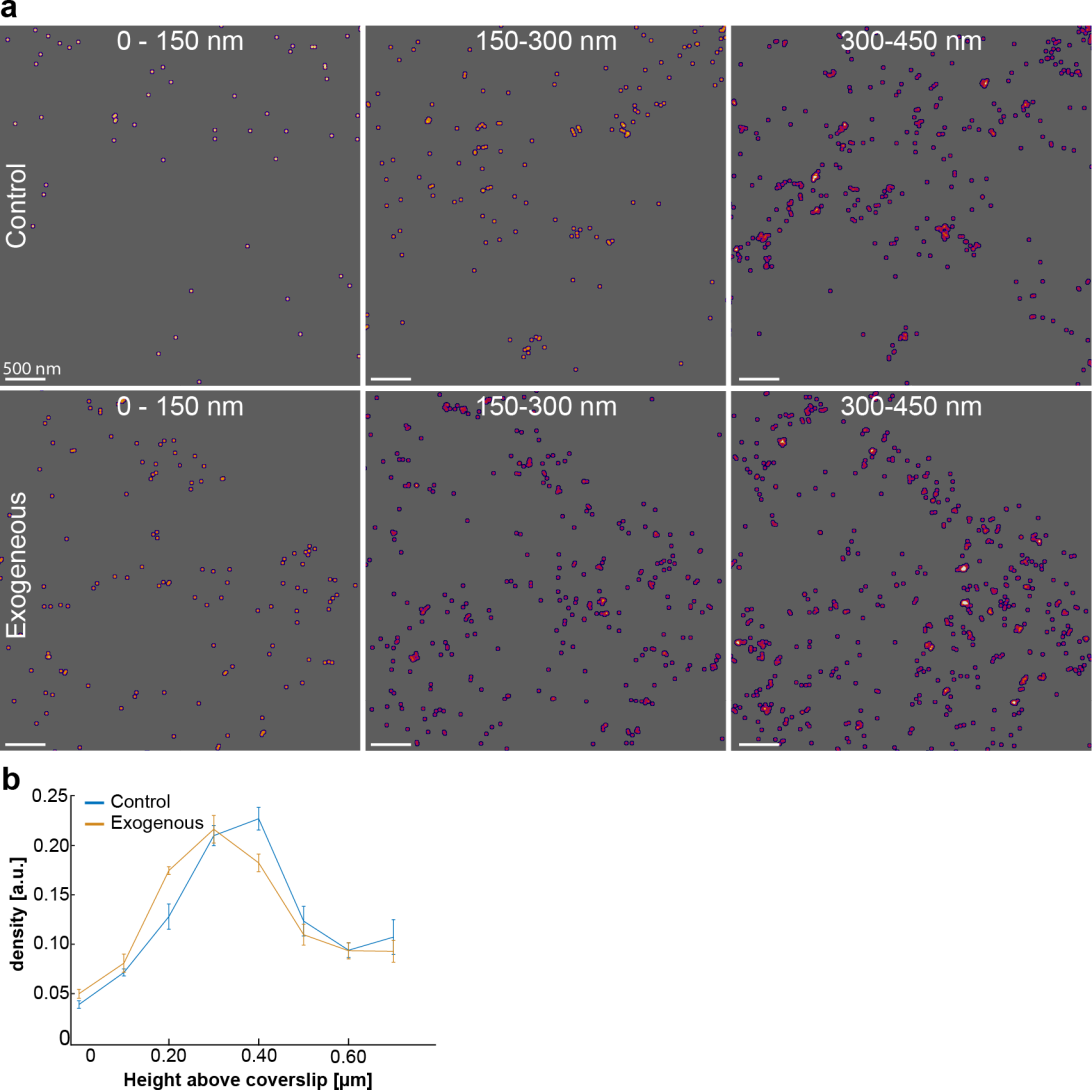
3D-STORM imaging of Na,K-ATPase β-subunit distribution. (a) 3D-STORM rendering of endogenous β1expression in the vicinity of the plasma membrane during control conditions or 18 hours after hNKAa1_SEP transfection. Each image shows the sum of events over 150 nm in z. (b) Normalized mean total surface density in 100 nm steps from plasma membrane into the center of the cell body, n = 4 or 5 cells, with both graphs individually normalized to yield a sum of 1.

Image analysis of PALM and STORM data was performed by the SMLocalizer plugin in Fiji [28]. SMLocalizer [29] is available online through http://smlocalizer.sourceforge.net/. In short: the raw data was corrected for background and drift, and then the localizations, including the precision, were calculated by nonlinear least square fit of the individual peaks to a 2D Gaussian. The precision for each peak was found by dividing the FWHM of the fitted Gaussian by the square root of the number of detected photons. Rendering was done by assigning pixel values based on number of events within each 5x5 nm pixel and smoothing the resulting image with a 10 nm sigma Gaussian to yield final images. The mean intensity within 5 regions within each image was used to compute the image mean distribution and the means of the image mean was normalized to control. Regions ranged in size from 0.7 to 2.5 μm^2^, exact size varied to avoid large voids of localizations and cell borders. For 3D-STORM localization, the built in 3D-PRILM algorithm, calibrated from an image stack of 40 nm fluorescent beads was applied to extract axial and lateral coordinates [27]. For cell images in Figs 1–3 the Fiji lookup table “Fire” was applied with the display value for 0 changed to [110,110,110].

### Pair correlation cluster analysis

Pair correlation cluster analysis was performed in Matlab following the protocol by Sengupta et. al. [30, 31]. In short, the autocorrelation, *g(r)*, was calculated in selected regions of each data set and then evaluated by fitting to models for random and nonrandom (i.e. clustered) protein distributions.

For random distribution the model was:

$$g\left(r\right)=g{\left(r\right)}^{stoch}+1=\frac{1}{4\pi \sigma^{2}\rho }\;exp\left(\frac{-r^{2}}{4\sigma^{2}}\right)+1$$

For nonrandom distribution the model was:

$$g\left(r\right)=g{\left(r\right)}^{stoch}+\left(A\;exp\left(\frac{-r}{\xi }\right)+1\right)*g{\left(r\right)}^{PSF}$$

$$g{\left(r\right)}^{PSF}=\frac{1}{4\pi \sigma^{2}}\;exp\left(\frac{-r^{2}}{4\sigma^{2}}\right)$$

where σ is the precision in the single molecule localization measurements, ρ is the average protein density and *r* is the radial distance. The fitting parameters A (amplitude) and *ξ* (the correlation length of clusters) were estimated by nonlinear least square fit.

### Statistics

Statistical analysis was performed using one-way analysis of variance (Anova) at p<0.05 significance level. All error bars depict standard error of mean.

## Results

Results from studies of overexpressed systems are not always easily interpreted as the quantity of the protein of interest often exceeds what the cell has been evolved to handle. Quantitative control in systems may however partly be alleviated as many proteins require interactions with other proteins for assembly to functional units. This assembly control may thus beneficially act to limit non-physiological (non-functional) overexpression. If moderate structural limitation is at hand, transient overexpression may still distort cellular structures, effects that must be considered when quantifying for example molecular numbers with single-molecule super-resolution microscopy [32, 33]. We have in this work selected to analyze the endogenous and competitive expression of the membrane bound Na,K-ATPase, a hetereomeric assembly with a catalytically active α-subunit that requires a β-subunit for full function and plasma membrane insertion [21]. By transient expression of the α1-subunit in Na,K-ATPase, we also wanted to investigate if endogenous levels of β1-subunits would rearrange from endogenous subunit assemblies to exogenous ones over time. Thus, allowing us to gain insight in how single-molecule quantitative analysis might be affected both as a function of competitive exogenous transfection and endogenous protein assembly processes.

### Endogenous vs exogenous α1 levels through STORM imaging

By using single-molecule direct STORM imaging [34], with a well characterized primary antibody [26], we established a baseline plasma membrane density of endogenous Na,K-ATP α_1_. Through use of total internal reflection fluorescence (TIRF) excitation we focused on the plasma membrane closest to the coverslip of the sample and calculated the mean number of localized events per unit area. In order to compare results from different protocols we chose to normalize this density to 100%. We then proceeded to study the effect of transfection of HA_hNKAa1_mMaple3 through immunostaining using antibodies against Na,K-ATP α_1_ and HA-tag. Samples were fixed after 17 or 41 hours of lipofectamine transfection applying standard amounts of plasmid DNA (see materials and methods). Single-molecule STORM analysis of samples immunostained for Na,K-ATP α_1_ revealed that the total Na,K-ATPase α_1_ density increased with 33% after 17 hours and 63% after 41 hours (Fig 1A and 1B). To verify that this increase comes from the transfected protein, we performed parallel STORM analysis of samples immunostained for HA-tag and found that the exogenous densities reached 38% respectively 79% of Na,K-ATP α_1_ control levels (Fig 1A and 1B).

In order to ascertain that transfecting a membrane protein did not change Na,K-ATPase α_1_ density we also transfected cells with a mock plasma membrane tether (syntaxin) fused to a tetracysteine motif (Cys–Cys–Xxx–Xxx–Cys–Cys) that binds FlAsH. Cells were labeled using FlAsH-EDT2 after 17 and 41 hours transfection and subsequently fixed and labeled using the antibody against Na,K-ATPase α_1_. Subsequent STORM imaging and quantification revealed no effect of mock transfection on Na, K-ATPase α_1_ density (Fig 1A and 1C).

Endogenous Na,K-ATPase α_1_ cluster size was evaluated using pair-correlation analysis by fitting the autocorrelation function for protein localizations to models for random and nonrandom protein distributions. The cluster size of the total population was analyzed for control, 17 h and 41 h after transfection (Fig 1D). All datasets fitted a model for nonrandom distribution, i.e. a clustered distribution. The cluster sizes, estimated as the fit parameter *ξ* (the correlation length), was 39±19 nm. The size of the clusters was uncorrelated to time after transfection and did not increase with the increased membrane protein density.

### Exogenous α1 levels through PALM imaging

By using single-molecule PALM imaging we proceeded to investigate the levels of exogenous Na,K-ATPase α_1_ incorporated into the plasma membrane during transfection using the photoconvertible mMaple3 as our reporter [24]. Using the same HA_hNKAa1_mMaple3 plasmid we fixed cells after 17 and 41 hours of transfection and proceeded to quantify plasma membrane α_1_ densities. Quantification shows a doubling (113% increase) between 17 and 41 hours (Fig 2), in line with what was found using STORM (108% increase, from 38% to 79% of control, see exogenous in Fig 1B). The mean precision, σ, was 20±5 nm.

### β1-subunit expression during α1 transfection

Previous studies suggest that functional assembly and membrane insertion of the heteromeric Na,K-ATPase assembly is regulated by availability of β-subunits [22, 23]. To analyze the potential role of β-subunits limiting the expression of Na,K-ATPase in the plasma membrane during overexpression of α1-subunits we performed 3D-STORM imaging of antibody labeled Na,K-ATPase β1 to probe the 3D-distribution in control and α1 overexpressed conditions (Fig 3). Transient transfection of hNKAa1_SEP was introduced as above for an overnight time of 18 hours. The volumetric β1 density in 100 nm thick sections, starting from the cell-coverslip interface and going up into the cell, was quantified and revealed that there is a cytoplasmic bulk of β1-subunits which is not depleted after transfection (Fig 3).

## Discussion

In this study we have analyzed the influence of protein overexpression during transient transfection by a quantification of protein densities using super-resolution STORM and PALM imaging. We found that there is a continuous increase in plasma membrane density of the exogenous protein, to some degree at the expense of the endogenous protein density. The plasma membrane density of Na,K-ATPase α1 increase with 63% after 41 h transfection, at which point almost half of the total density is due to exogenous expression (Fig 1). The analysis shows that the endogenous component decreased by 16% under these conditions. In other words, the imaging shows that there is a capacity to increase Na,K-ATPase α1 membrane densities well above the endogenous control levels during transient transfection and that there is competition between endogenous and exogenous expression.

The data fit a model for competition between endogenous and exogenous expression. The half-life for Na,K-ATPase α1 in the membrane is 40 hours [35]. The density *D(t)* of a membrane protein produced at rate *P* and degraded at rate *k*, (*k = ln(2)/40)*, can be described by:

$$D\left(t\right)=\left(1-\frac{P}{k}\right)e^{-kt}+\frac{P}{k}$$

In nontransfected cells under steady state, *P/k = 1 and D(t) = 1*. Solving this equation for D(41) = 1.63 (63% increase of total α1 at 41 hours, cf. total in Fig 1B) give a production/degradation rate *P/k*≈2.24, which is the sum of endogenous and exogenous expression.

If the endogenous expression was not reduced by competition from exogenous expression, the total membrane density would have increased by 79% at 41 h after transfection (cf. exogenous in Fig 1B), corresponding to a production/degradation rate *P/k*≈2.55. The difference in production/degradation rates is then likely due to a competition over factors that limit the expression and insertion of Na,K-ATPase in the membrane. This can be seen as a reduced expression of the endogenous protein by competitive exogenous expression.

Of interest here is the observation that there is a relatively large intracellular bulk of β1 subunits remaining after overexpression of α1 (Fig 3), which suggest that there are other mechanisms than only the availability of beta subunits that regulate and limit the Na,K-ATPase plasma membrane insertion. Those mechanisms remain to be identified.

We have here used single-molecule localization microscopy (SMLM) techniques that generate super-resolution images by serially detecting individual fluorescent molecules. The power of SMLM goes beyond images: biologically relevant information can be extracted from the mathematical relationships between the positions of the fluorophores in space and time [36]. Here we demonstrate the use of SMLM for an analysis of membrane protein overexpression in terms of both density and spatial clustering. With the imaging-based approach we find that Na,K-ATPase can be found in clusters in the membrane but also that an increase in Na,K-ATPase membrane protein density is not accompanied by an increased size of those protein clusters. It cannot be ruled out that the observed clusters are a post-fixation effect. Tanaka et.al. [37] have demonstrated that conventional fixation protocols based on paraformaldehyde or methanol do not always completely immobilize membrane proteins and that artificial clusters of membrane proteins can be formed by diffusion and antibody crosslinking.

The type of combined spatial and quantitative observations we report here are not possible to directly obtain with conventional methods for estimation of membrane protein densities, such as cell surface biotinylation or subcellular fractionation followed by quantitative immunoblotting.

Tight control of the protocols used for fluorescent labeling is necessary when striving to report reliable molecular quantification numbers based on SMLM methods. It is worth noting that quantification based on PALM or STORM imaging give differences in absolute plasma membrane densities (cf. total number of localized events in Figs 1 and 2). In the PALM imaging protocol all relevant proteins can be assumed to be fluorescently tagged, but the fluorescent protein conversion rate comes into play for quantification [24]. Absolute numbers reported by PALM imaging can thus be considered as an underestimate, while numbers reported by STORM imaging may, due to frequent fluorescent dye blinking, be considered as an overestimate. In STORM imaging, the antibody specificity and affinity are important factors [16]. A possible difference in affinity of the two antibodies used may affect the estimate of exogenous protein vs endogenous protein density. To minimize this influence, we selected well characterized and highly specific monoclonal antibodies and used them in excess concentration for an efficient and uniform labeling. Furthermore, we used the same fluorescent probe (Alexa 647) in all STORM experiments to make sure that blinking behavior was constant.

Both STORM and PALM imaging methods are thus internally consistent over time, which allows for intra-method direct comparisons and inter-method normalized comparisons to be done.

## Conclusions

Taken together, our data illustrate that there are considerations that have to be taken into account when performing studies using transient transfections and overexpression of proteins. Of importance is that under most conditions, cells will express a combination of endogenous and exogenous proteins, which might not be under quantitative control. If exogenous expression is made in order to study a mutant form of the protein, the functional readouts will be a sum of both endogenous and exogenous effects. If the exogenous protein is tagged for imaging or interaction studies, the non-imaged component from endogenous proteins will need to be taken into account. This calls for control experiments in non-transfected systems to understand to what extent the factor being investigated is affected by introduction of the exogenous protein. SMLM techniques are thus suitable for both quantitative characterization and to probe endogenous and exogenous translation.

## References

[1] Betzig E, Patterson GH, Sougrat R, Lindwasser OW, Olenych S, Bonifacino JS, et al Imaging Intracellular Fluorescent Proteins at Nanometer Resolution. Science. 2006;313(5793):1642 doi: 10.1126/science.1127344 16902090 10.1126/science.1127344

[2] Hess ST, Girirajan TPK, Mason MD. Ultra-High Resolution Imaging by Fluorescence Photoactivation Localization Microscopy. Biophysical Journal. 2006;91(11):4258–72. doi: 10.1529/biophysj.106.091116 16980368 10.1529/biophysj.106.091116 PMC1635685

[3] Rust MJ, Bates M, Zhuang X. Sub-diffraction-limit imaging by stochastic optical reconstruction microscopy (STORM). Nat Meth. 2006;3(10):793–6. doi: 10.1038/nmeth929 16896339 10.1038/nmeth929 PMC2700296

[4] Egner A, Geisler C, von Middendorff C, Bock H, Wenzel D, Medda R, et al Fluorescence Nanoscopy in Whole Cells by Asynchronous Localization of Photoswitching Emitters. Biophysical Journal. 2007;93(9):3285–90. doi: 10.1529/biophysj.107.112201 17660318 10.1529/biophysj.107.112201 PMC2025649

[5] Heilemann M, van de Linde S, Schüttpelz M, Kasper R, Seefeldt B, Mukherjee A, et al Subdiffraction-Resolution Fluorescence Imaging with Conventional Fluorescent Probes. Angewandte Chemie International Edition. 2008;47(33):6172–6. doi: 10.1002/anie.200802376 18646237 10.1002/anie.200802376

[6] Huang B, Wang W, Bates M, Zhuang X. Three-Dimensional Super-Resolution Imaging by Stochastic Optical Reconstruction Microscopy. Science. 2008;319(5864):810 doi: 10.1126/science.1153529 18174397 10.1126/science.1153529 PMC2633023

[7] Juette MF, Gould TJ, Lessard MD, Mlodzianoski MJ, Nagpure BS, Bennett BT, et al Three-dimensional sub-100 nm resolution fluorescence microscopy of thick samples. Nat Meth. 2008;5(6):527–9. doi: 10.1038/nmeth.1211 18469823 10.1038/nmeth.1211

[8] Lando D, Endesfelder U, Berger H, Subramanian L, Dunne PD, McColl J, et al Quantitative single-molecule microscopy reveals that CENP-A(Cnp1) deposition occurs during G2 in fission yeast. Open Biology. 2012;2(7):120078 doi: 10.1098/rsob.120078 22870388 10.1098/rsob.120078 PMC3411111

[9] Puchner EM, Walter JM, Kasper R, Huang B, Lim WA. Counting molecules in single organelles with superresolution microscopy allows tracking of the endosome maturation trajectory. Proceedings of the National Academy of Sciences of the United States of America. 2013;110(40):16015–20. doi: 10.1073/pnas.1309676110 24043832 10.1073/pnas.1309676110 PMC3791776

[10] Nan X, Collisson EA, Lewis S, Huang J, Tamgüney TM, Liphardt JT, et al Single-molecule superresolution imaging allows quantitative analysis of RAF multimer formation and signaling. Proceedings of the National Academy of Sciences of the United States of America. 2013;110(46):18519–24. doi: 10.1073/pnas.1318188110 24158481 10.1073/pnas.1318188110 PMC3831949

[11] Ehmann N, van de Linde S, Alon A, Ljaschenko D, Keung XZ, Holm T, et al Quantitative super-resolution imaging of Bruchpilot distinguishes active zone states. 2014;5:4650 doi: 10.1038/ncomms5650 25130366 10.1038/ncomms5650 PMC4143948

[12] Coltharp C, Kessler RP, Xiao J. Accurate Construction of Photoactivated Localization Microscopy (PALM) Images for Quantitative Measurements. PLoS One. 2012;7(12):e51725 doi: 10.1371/journal.pone.0051725 23251611 10.1371/journal.pone.0051725 PMC3520911

[13] Shivanandan A, Deschout H, Scarselli M, Radenovic A. Challenges in quantitative single molecule localization microscopy. FEBS Letters. 2014;588(19):3595–602. doi: 10.1016/j.febslet.2014.06.014 24928440 10.1016/j.febslet.2014.06.014

[14] Durisic N, Cuervo LL, Lakadamyali M. Quantitative super-resolution microscopy: pitfalls and strategies for image analysis. Current Opinion in Chemical Biology. 2014;20:22–8. doi: 10.1016/j.cbpa.2014.04.005 24793374 10.1016/j.cbpa.2014.04.005

[15] Fricke F, Beaudouin J, Eils R, Heilemann M. One, two or three? Probing the stoichiometry of membrane proteins by single-molecule localization microscopy. 2015;5:14072 doi: 10.1038/srep14072 26358640 10.1038/srep14072 PMC4642553

[16] Opazo F, Levy M, Byrom M, Schafer C, Geisler C, Groemer TW, et al Aptamers as potential tools for super-resolution microscopy. Nat Meth. 2012;9(10):938–9. doi: 10.1038/nmeth.2179 23018995 10.1038/nmeth.2179

[17] Ries J, Kaplan C, Platonova E, Eghlidi H, Ewers H. A simple, versatile method for GFP-based super-resolution microscopy via nanobodies. Nat Meth. 2012;9(6):582–4. doi: 10.1038/nmeth.1991 22543348 10.1038/nmeth.1991

[18] Ratz M, Testa I, Hell SW, Jakobs S. CRISPR/Cas9-mediated endogenous protein tagging for RESOLFT super-resolution microscopy of living human cells. Scientific Reports. 2015;5:9592 doi: 10.1038/srep09592 25892259 10.1038/srep09592 PMC4402611

[19] Gibson TJ, Seiler M, Veitia RA. The transience of transient overexpression. Nat Meth. 2013;10(8):715–21. doi: 10.1038/nmeth.2534 23900254 10.1038/nmeth.2534

[20] Violin JD, DeWire SM, Barnes WG, Lefkowitz RJ. G Protein-coupled Receptor Kinase and β-Arrestin-mediated Desensitization of the Angiotensin II Type 1A Receptor Elucidated by Diacylglycerol Dynamics. Journal of Biological Chemistry. 2006;281(47):36411–9. doi: 10.1074/jbc.M607956200 17008309 10.1074/jbc.M607956200

[21] Ceroni F, Algar R, Stan G-B, Ellis T. Quantifying cellular capacity identifies gene expression designs with reduced burden. Nat Meth. 2015;12(5):415–8. doi: 10.1038/nmeth.3339 25849635 10.1038/nmeth.3339

[22] Ackermann U, Geering K. Mutual dependence of Na,K-ATPase α- and β-subunits for correct posttranslational processing and intracellular transport. FEBS Letters. 1990;269(1):105–8. doi: 10.1016/0014-5793(90)81130-G 2167238 10.1016/0014-5793(90)81130-g

[23] Tokhtaeva E, Sachs G, Vagin O. Assembly with the Na,K-ATPase α(1) Subunit Is Required for Export of β(1) and β(2) Subunits from the Endoplasmic Reticulum. Biochemistry. 2009;48(48):11421–31. doi: 10.1021/bi901438z 19764716 10.1021/bi901438z PMC2987690

[24] Wang S, Moffitt JR, Dempsey GT, Xie XS, Zhuang X. Characterization and development of photoactivatable fluorescent proteins for single-molecule–based superresolution imaging. Proceedings of the National Academy of Sciences. 2014;111(23):8452–7. doi: 10.1073/pnas.1406593111 24912163 10.1073/pnas.1406593111 PMC4060684

[25] Liebmann T, Kruusmägi M, Sourial-Bassillious N, Bondar A, Svenningsson P, Flajolet M, et al A Noncanonical Postsynaptic Transport Route for a GPCR Belonging to the Serotonin Receptor Family. The Journal of Neuroscience. 2012;32(50):17998–8008. doi: 10.1523/JNEUROSCI.1804-12.2012 23238716 10.1523/JNEUROSCI.1804-12.2012 PMC6621742

[26] Arystarkhova E, Sweadner KJ. Isoform-specific Monoclonal Antibodies to Na,K-ATPase α Subunits: Evidence for a tissue-specific post-translational modification of the alpha subunit. Journal of Biological Chemistry. 1996;271(38):23407–17. doi: 10.1074/jbc.271.38.23407 8798546 10.1074/jbc.271.38.23407

[27] Baddeley D, Cannell MB, Soeller C. Three-dimensional sub-100 nm super-resolution imaging of biological samples using a phase ramp in the objective pupil. Nano Research. 2011;4(6):589–98. doi: 10.1007/s12274-011-0115-z

[28] Schindelin J, Arganda-Carreras I, Frise E, Kaynig V, Longair M, Pietzsch T, et al Fiji: an open-source platform for biological-image analysis. Nat Meth. 2012;9(7):676–82. doi: 10.1038/nmeth.2019 22743772 10.1038/nmeth.2019 PMC3855844

[29] Bernhem K, Brismar H. SMLocalizer, a GPU accelerated ImageJ plugin for single molecule localization microscopy. Bioinformatics. 2018;34(1):137–8. doi: 10.1093/bioinformatics/btx553 28968783 10.1093/bioinformatics/btx553 PMC5870682

[30] Sengupta P, Jovanovic-Talisman T, Skoko D, Renz M, Veatch SL, Lippincott-Schwartz J. Probing protein heterogeneity in the plasma membrane using PALM and pair correlation analysis. Nat Methods. 2011;8(11):969–975. doi: 10.1038/nmeth.1704 21926998 10.1038/nmeth.1704 PMC3400087

[31] Sengupta P, Jovanovic-Talisman T, Lippincott-Schwartz J. Quantifying spatial organization in point-localization superresolution images using pair correlation analysis. Nat Protoc. 2013;8(2):345–354. doi: 10.1038/nprot.2013.005 23348362 10.1038/nprot.2013.005 PMC3925398

[32] Nair D, Hosy E, Petersen JD, Constals A, Giannone G, Choquet D, et al Super-Resolution Imaging Reveals That AMPA Receptors Inside Synapses Are Dynamically Organized in Nanodomains Regulated by PSD95. The Journal of Neuroscience. 2013;33(32):13204–24. doi: 10.1523/JNEUROSCI.2381-12.2013 23926273 10.1523/JNEUROSCI.2381-12.2013 PMC6619720

[33] MacGillavry HD, Song Y, Raghavachari S, Blanpied TA. Nanoscale scaffolding domains within the postsynaptic density concentrate synaptic AMPA receptors. Neuron. 2013;78(4):615–22. doi: 10.1016/j.neuron.2013.03.009 23719161 10.1016/j.neuron.2013.03.009 PMC3668352

[34] van de Linde S, Loschberger A, Klein T, Heidbreder M, Wolter S, Heilemann M, et al Direct stochastic optical reconstruction microscopy with standard fluorescent probes. Nat Protocols. 2011;6(7):991–1009. doi: 10.1038/nprot.2011.336 21720313 10.1038/nprot.2011.336

[35] Tamkun Michael M., Fambrough Douglas M. The (Na^+^ + K^+^)-ATPase of chick sensory neurons. Studies on biosynthesis and intracellular transport. J Biol Chem. 1986;261(3):1009–19. http://www.jbc.org/content/261/3/1009.full.pdf 3003048

[36] Nicovich Philip R, Owen Dylan M& Gauss Katharina. Turning single-molecule localization microscopy into a quantitative bioanalytical tool. Nature Protocols 2017;12:453–460. doi: 10.1038/nprot.2016.166 28151466 10.1038/nprot.2016.166

[37] Tanaka KAK, Suzuki KGN, Shirai YM, Shibutani ST, Miyahara MSH, Tsuboi H, et al Membrane molecules mobile even after chemical fixation. Nat Methods 2010;7: 865–866. doi: 10.1038/nmeth.f.314 20881966 10.1038/nmeth.f.314

